# Undifferentiated spermatogonia modulate their behavior via the expression of basement membrane protein laminin^†^

**DOI:** 10.1093/biolre/ioag032

**Published:** 2026-02-03

**Authors:** Yusuke Kawabe, Saya Yamada, Yuichi Shima, Kentaro Tanemura, Shosei Yoshida, Kenshiro Hara

**Affiliations:** Laboratory of Animal Reproduction and Development, Graduate School of Agricultural Science, Tohoku University, 468-1 Aoba, Aramaki-aza, Aoba-ku, Sendai, Miyagi 980-8572, Japan; Laboratory of Animal Reproduction and Development, Graduate School of Agricultural Science, Tohoku University, 468-1 Aoba, Aramaki-aza, Aoba-ku, Sendai, Miyagi 980-8572, Japan; Division of Microscopic and Developmental Anatomy, Department of Anatomy, Kurume University School of Medicine, 67 Asahi-machi, Kurume, Fukuoka 830-0011, Japan; Laboratory of Animal Reproduction and Development, Graduate School of Agricultural Science, Tohoku University, 468-1 Aoba, Aramaki-aza, Aoba-ku, Sendai, Miyagi 980-8572, Japan; Division of Germ Cell Biology, National Institute for Basic Biology, National Institutes of Natural Sciences, 5-1 Higashiyama, Myodaiji, Okazaki, Aichi 444-8787, Japan; Graduate Institute for Advanced Studies, SOKENDAI, 5-1 Higashiyama, Myodaiji, Okazaki, Aichi 444-8787, Japan; Laboratory of Animal Reproduction and Development, Graduate School of Agricultural Science, Tohoku University, 468-1 Aoba, Aramaki-aza, Aoba-ku, Sendai, Miyagi 980-8572, Japan; Advanced Research Division for New Fields within a higher research organization, Tohoku University, Japan

**Keywords:** extracellular matrix, spermatogonia, testicular basement membrane, conditional knockout, laminin, mouse

## Abstract

In the mouse testis, spermatogonial stem cells (SSCs) are sparsely distributed and migrate along the basement membrane of seminiferous tubules. Although the basement membrane is generally thought to be formed by surrounding somatic cells, whether SSCs also produce basement membrane proteins and, if so, whether SSC-produced laminin affects SSC behavior remains unknown. In this study, we found that mouse GFRα1^+^ spermatogonia, which include SSCs, expressed several laminin subunit genes, including *Lamc1*, whose expression declined upon differentiation. To test whether GFRα1^+^ spermatogonia-derived laminin regulates their behavior, we used two conditional knockout mouse models. In the Vasa-Cre model, which induces recombination in all germ cells, heterozygous deletion of *Lamc1* increased both cell death and proliferation of GFRα1^+^ spermatogonia, while maintaining an apparent steady state of GFRα1^+^ cell density and spermatogenesis. In the tamoxifen-inducible GFRα1-CreER model carrying *Lamc1^flox/flox^*, tamoxifen-induced *Lamc1* deletion in GFRα1^+^ spermatogonia caused a rapid reduction in their cell density within a few days, followed by increased proliferation and an imbalance between proliferation and differentiation of GFRα1^+^ spermatogonia that led to the restoration of GFRα1^+^ spermatogonial density. Collectively, these genetic findings suggest that GFRα1^+^ spermatogonia modulate their survival and behavior through laminin expression, likely by influencing the basement membrane around GFRα1^+^ spermatogonia. Such cell-autonomous regulation allows GFRα1^+^ spermatogonia, including SSCs, to form an appropriate local microenvironment wherever they reside within the testicular open niche, supporting stable behavior of spermatogonia during spermatogenesis.

## Teaser

In the mouse testis, GFRα1^+^ spermatogonia, including spermatogonial stem cells, modulate their own survival and behavior by expressing Laminin, which likely contributes to forming the local microenvironment surrounding them within the open niche.

## Graphical Abstract

**Graphical Abstract:**
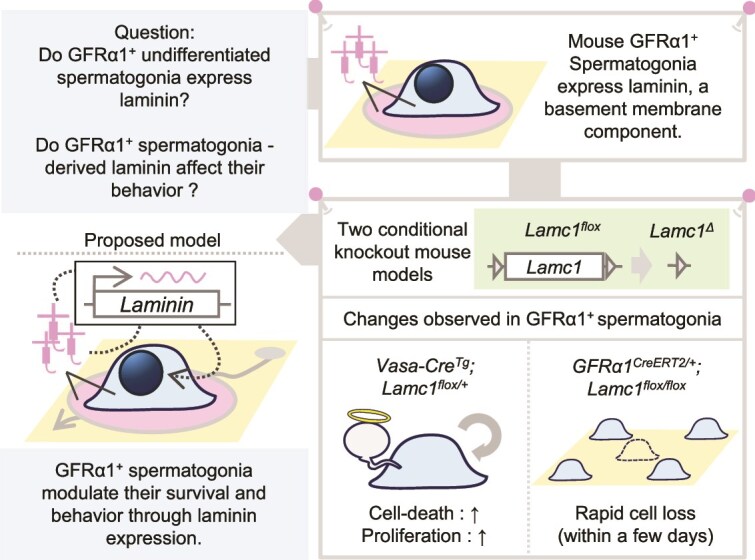

## Introduction

**Figure 1:**
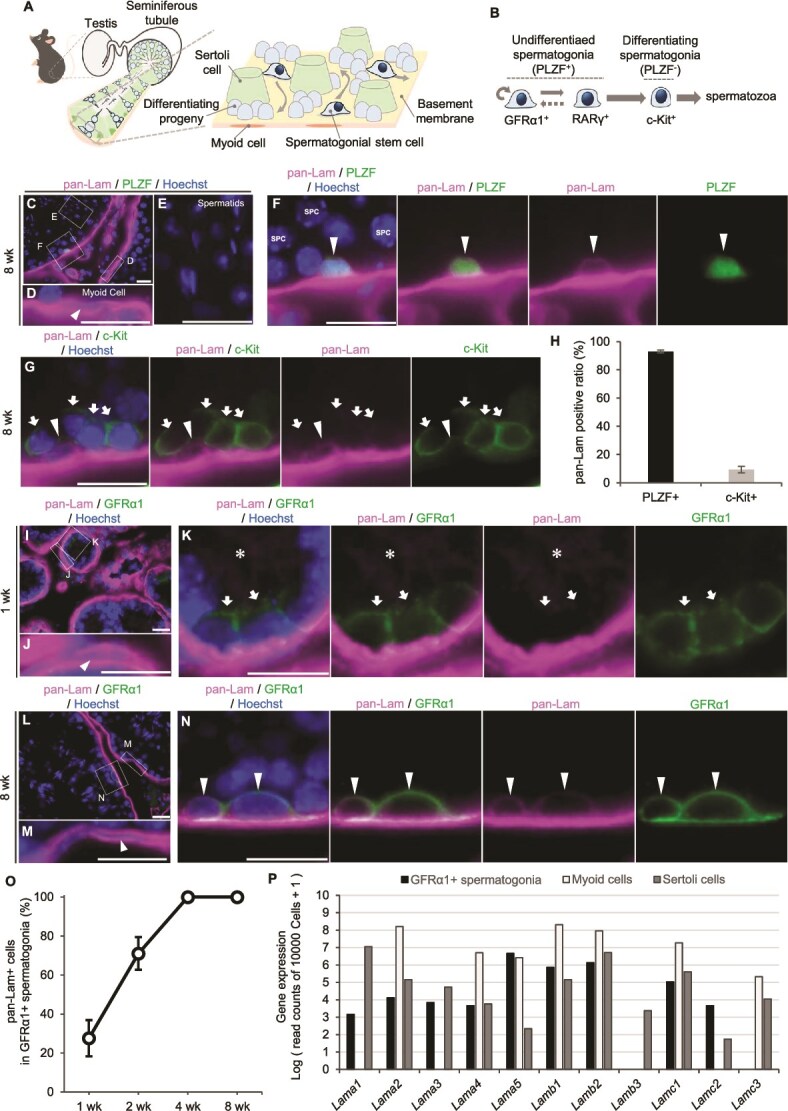
Laminin expression in GFRα1^+^ spermatogonia. (A) Schematic of seminiferous tubules in the mouse testis. Spermatogonial stem cells are sparsely distributed and dynamically migrate among Sertoli cells and differentiating progeny on the basement membrane of seminiferous tubules. (B) Subpopulation of spermatogonia classified by gene expression. GFRα1^+^ and RARγ^+^ cells correspond to A_undiff_ that are PLZF^+^, whereas c-Kit^+^ cells correspond to differentiating spermatogonia that are PLZF^−^. A circular arrow indicates self-renewal, the rightward arrow between GFRα1^+^ and RARγ^+^ spermatogonia indicates transition, the other rightward arrows indicate differentiation, and the dashed arrow indicates infrequent reversion events [4]. (C–F) Testis sections from 8-week-old mice immunostained for pan-Laminin (magenta) and PLZF (green), with nuclei counterstained by Hoechst33342 (blue). Panels (D–F) show higher magnified views of the boxed regions in (C). An arrowhead in (D) indicates a pan-Laminin^+^ peritubular myoid cell. Spermatids (E) and spermatocytes (SPC, F) are negative for pan-Laminin. An arrowhead in (F) indicates a pan-Laminin^+^ /PLZF^+^ spermatogonium. (G) Testis sections from 8-week-old mice immunostained for pan-Laminin (magenta) and c-Kit (green). An arrowhead indicates pan-Laminin^+^/c-Kit^−^ spermatogonium, and arrows indicate pan-Laminin^−^/c-Kit^+^ spermatogonia. (H) Proportion of pan-Laminin^+^ cells among PLZF^+^ or c-Kit^+^ spermatogonia in 8-week-old testes. PLZF^+^ and c-Kit^+^ spermatogonia were counted in 50 seminiferous tubule sections, respectively (*n* = 3 individuals). (I–N) pan-Laminin (magenta) and GFRα1 (green) immunostaining of testes from 1-week-old (I-K) and 8-week-old (L-N) mice. (J, K) and (M, N) are enlarged views of the boxed regions in (I) and (L), respectively. Arrowheads in (J, M) indicate pan-Laminin^+^ peritubular myoid cells. White asterisks in (K) indicate expression in the pan-Laminin^+^ Sertoli cells. Arrows in (K) indicate pan-Laminin^−^/GFRα1^+^ spermatogonia, while arrowheads in (N) indicate pan-Laminin^+^/GFRα1^+^ spermatogonia. (O) Percentage of pan-Laminin^+^ cells among GFRα1^+^ spermatogonia at 1, 2, 4, and 8 weeks of age. GFRα1^+^ spermatogonia were counted in 50 seminiferous tubules sections per individual (*n* = 3 individuals at each time point). (P) Bar graphs showing laminin subunit gene expression levels in GFRα1^+^ spermatogonia, myoid cells, and Sertoli cells from adult mouse testes, based on a published single-cell RNA-seq dataset [5]. Data are presented as mean ± SD. Scale bars, 20 μm.

In mammals, spermatogonial stem cells (SSCs) are responsible for the lifelong maintenance of spermatogenesis by balancing proliferation and differentiation [1] and can regenerate spermatogenesis after germ cell loss [2, 3]. Within the seminiferous tubules, SSCs reside on the basement membrane. The SSC niche appears to lack a discrete anatomical structure and is therefore often described as an open (or facultative) niche. In this niche, SSCs are sparsely distributed and migrate dynamically among Sertoli cells and differentiating progeny (Figure 1A) [6, 7]. Therefore, unlike closed (or definitive) niches with discrete and invariant anatomical structures, such as *Drosophila* germline stem cell niche, the local microenvironment surrounding each SSC is not spatially fixed [1, 8, 9]. Although this microenvironment is likely to be organized mainly by somatic cells (e.g., peritubular myoid cells, Sertoli cells and lymphatic endothelial cells) [10–12], SSCs might also contribute to its organization to regulate their own behavior.

In mice, SSC function is included within undifferentiated spermatogonia (A_undiff_) [7]. A_undiff_ express α6-integrin, β1-integrin, and PLZF, and are morphologically classified into A_single_ (A_s_), A_paired_ (A_pr_), and A_aligned_ (A_al_) [13–17]. Among these, GFRα1^+^ spermatogonia comprise mainly A_s_ and A_pr_, and to a lesser extent A_al-4_ syncytia; this population includes SSCs and exhibits migratory behavior while supporting steady-state spermatogenesis [6, 18]. GFRα1^+^ spermatogonia give rise to Ngn3^+^/RARγ^+^ spermatogonia, an undifferentiated spermatogonial subset primed for differentiation, which subsequently differentiate into c-Kit^+^ differentiating spermatogonia and eventually into spermatozoa (Figure 1B) [4, 19, 20].

In general, a key component of the stem cell niche is the extracellular matrix (ECM), including basement membrane proteins such as collagen, fibronectin, and laminin [21–23]. In the hair follicle, the basement membrane provides both pericellular structural and signaling support to stem cells [24]. However, in the mouse testis, the basement membrane of seminiferous tubules has been regarded as a passive scaffold produced by surrounding somatic cells, particularly peritubular myoid cells, with limited evidence for its active involvement in SSC regulation [25–27]. Among the basement membrane components, laminin, a major ECM protein composed of α, β, and γ subunits [28–30], provides structural support and transmits signals through receptors such as α6β1 integrin, which is expressed in A_undiff_ [15]. Laminin in the seminiferous tubules has long been thought to be secreted mainly by peritubular myoid cells and Sertoli cells [12, 31]. Intriguingly, however, immunoelectron microscopy of rat testes has revealed the presence of laminin-positive secretory vesicles within spermatogonia [31], raising the possibility that SSCs themselves may also produce laminin to influence their local microenvironment. Nevertheless, whether SSCs indeed express laminin subunit genes and whether such expression functionally affects SSC behavior remains largely unexplored.

In this study, we analyzed laminin expression in GFRα1^+^ spermatogonia in adult mouse testis and found that they express laminin subunit genes, including *Lamc1*. To test whether spermatogonia-derived laminin regulates spermatogonial behavior, we employed two distinct conditional knockout (cKO) mouse models. First, germline heterozygous *Lamc1* cKO mice (*Vasa-Cre^Tg^; Lamc1^flox/+^*) were used to assess the long-term effects of reduced *Lamc1* dosage in germ cells. Second, tamoxifen-inducible GFRα1^+^ spermatogonia-specific homozygous *Lamc1* cKO mice (*GFRα1^CreERT2/+^; Lamc1^flox/flox^*) were used to evaluate the short-term effects of acute *Lamc1* loss specifically in GFRα1^+^ spermatogonia.

## Methods

### Animals

*Vasa-Cre^Tg^* mice ([32]; #006954, Jackson Laboratory, Bar Harbor, ME, USA), *GFRα1^CreERT2^* mice [33], *Lamc1^flox^* mice [34], and *CAG-CAT-EGFP^Tg^* mice [35] were used as described previously. *Vasa-Cre* mice were maintained on a FVB/NJ background and crossed twice with C57BL/6J mice before use in experiments. All other strains were maintained on the C57BL/6J background. Wild-type C57BL/6J mice were purchased from Japan SLC, Inc. (Shizuoka, Japan). All mice were housed in a 12-h light/dark cycle, with free access to food (MF, Oriental Yeast Co., Ltd., Tokyo, Japan) and water. The animals were defined as adult mice at 8–9 weeks of age, unless otherwise noted. All animal experiments were approved by the Tohoku University Institutional Animal Care and Use Committee (2020AgA-012-02; 2021AgA-005-02; 2023AgA-001).

### Primers and polymerase chain reaction conditions

Genotyping was performed using newly designed and previously reported primers. The primer set for *Lamc1* exon2 full-length detection (F: 5′-CCTCACGAGCACAGACGAAA-3′, R: 5′-AATGTTACTGGACGTCCCCAGAG-3′) was used to identify the *Lamc1^+^* (1.1 kbp), *Lamc1^flox^* (3.0 kbp), and *Lamc1^Δ^* (2.1 kbp) alleles. A second primer set for *Lamc1* flox (F:5′-CCTCACGAGCACAGACGAAA-3′, R:5′-GGGTGGCTAGGTTTTAATGCCA-3′) was used to detect the *Lamc1^flox^* (371 bp) and *Lamc1^+^* (337 bp) alleles.

Polymerase chain reaction (PCR) for the full-length *Lamc1* allele was carried out using Takara LA Taq (Takara Bio Inc., Shiga, Japan: Cat. RR002A), with the following conditions: an initial denaturation at 94°C for 5 min; eight touchdown cycles (94°C for 30 s, 68°C for 30 s with a 1°C decrease per cycle, and 72°C for 5 min); 31 cycles of amplification (94°C for 30 s, 60°C for 30 s, and 72°C for 5 min); and a final extension at 72°C for 7 min.

For the *Lamc1*^flox^ and other alleles, including *GFRα1^CreERT2^* (300 bp: [6]), *CAG-CAT-EGFP^Tg^* (360 bp: [35]), internal positive control (IPC: 200 bp: oIMR8744 and oIMR8745, Jackson Laboratory), *Vasa-Cre^Tg^* (240 bp: [32], oIMR7643 and oIMR7644, Jackson Laboratory), PCR was performed using Takara Taq (Takara Bio Inc, Cat. #RR001A) under the following conditions: an initial denaturation at 94°C for 5 min; eight touchdown cycles (94°C for 20 s, 68°C for 30 s with a 1°C decrease per cycle, and 72°C for 30 s); 27 cycles of amplification (94°C for 20 s, 60°C for 30 s, and 72°C for 30 s); and a final extension at 72°C for 5 min.

### *Lamc1* conditional knockout using *Vasa-Cre*^*Tg*^

**Figure 2:**
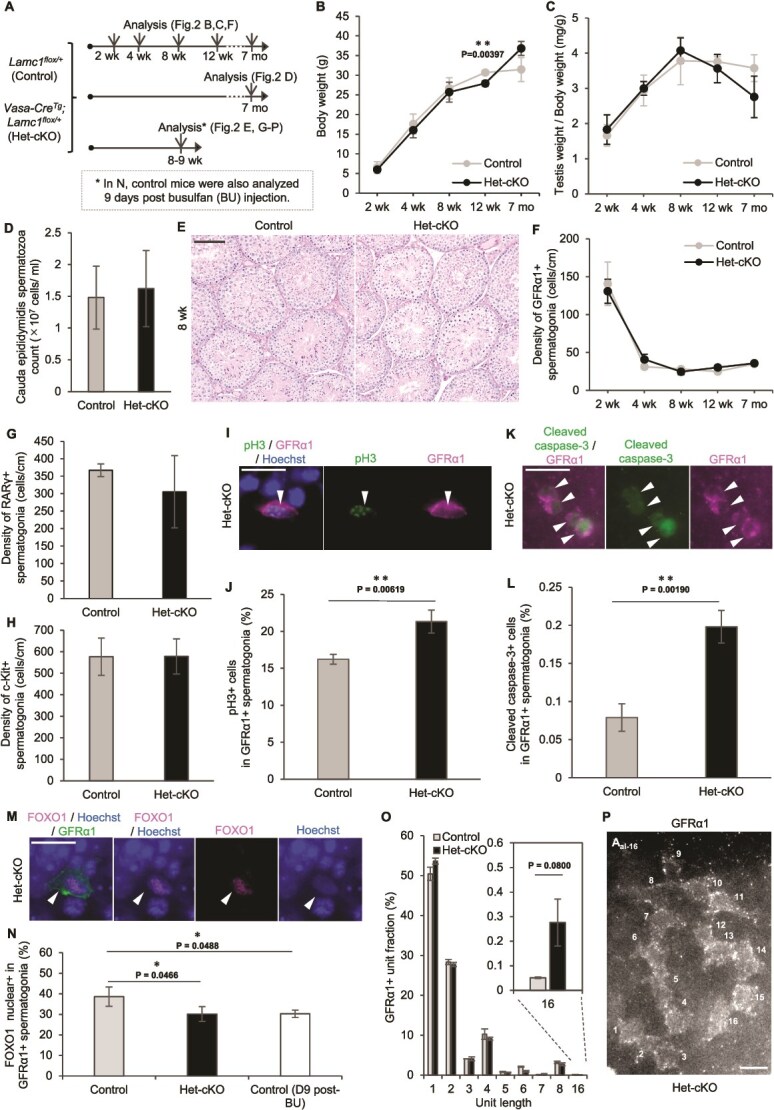
Phenotype of GFRα1^+^ spermatogonia in the *Vasa-Cre^Tg^; Lamc1^flox/+^* mouse testes. (A) Experimental schedule using *Vasa-Cre^Tg^; Lamc1^flox/+^* mice (Het-cKO) and *Lamc1^flox/+^* (Control) mice. (B, C) Body weight (B) and testis weight normalized to body weight (C) in Control and Het-cKO mice at different ages. (D) Cauda epididymal sperm counts in Control and Het-cKO mice at 7 months. (E) PAS-hematoxylin-stained cross-sections of Control and Het-cKO testes at 8 weeks. (F) Relative density of GFRα1^+^ spermatogonia along the seminiferous tubule perimeter in Control and Het-cKO mice at different ages. GFRα1^+^ spermatogonia were counted in 50 seminiferous tubule sections per individual at 2 and 4 weeks, and in 90 sections at 8 and 12 weeks and 7 months (*n* = 3 individuals per time point). (G, H) Density of RARγ^+^ (G) and c-Kit^+^ (H) spermatogonia along the seminiferous tubule perimeter in Control and Het-cKO mice at 8–9 weeks. RARγ^+^ and c-Kit^+^ spermatogonia were counted in 30 seminiferous tubule sections (*n* = 3 individuals). (I, J) Immunofluorescent staining of testis sections for GFRα1 (magenta) and pH3 (green), with Hoechst33342 staining (blue), in Het-cKO testis at 8–9 weeks. Arrowhead indicates a pH3^+^/GFRα1^+^ spermatogonium in Het-cKO (I). The proportion of pH3^+^ cells among GFRα1^+^ spermatogonia in the Control and Het-cKO testes is shown in (J). GFRα1^+^ spermatogonia were counted in 90 seminiferous tubules sections per individual (*n* = 3 individuals). (K, L) Whole-mount immunofluorescent staining for cleaved caspase-3 (green) and GFRα1 (magenta) in Het-cKO testis at 8–9 weeks. Arrowheads indicate cleaved caspase-3^+^/GFRα1^+^ spermatogonia in Het-cKO (K). The percentage of cleaved caspase-3^+^ cells among GFRα1^+^ spermatogonia in the Control and Het-cKO testes is shown in (L). (M, N) Whole-mount immunofluorescent staining for GFRα1 (green) and FOXO1 (magenta), with Hoechst33342 staining (blue), in Het-cKO testes at 8–9 weeks. Arrowhead in (M) indicates a GFRα1^+^ spermatogonium with a nuclear FOXO1^+^ signal. The proportion of GFRα1^+^ spermatogonia with nuclear FOXO1 in Control, Het-cKO, and busulfan-treated is shown in (N). (O, P) Frequency of GFRα1^+^ spermatogonia classified by syncytial length in Control and Het-cKO testes at 8–9 weeks (O). Representative whole-mount immunostained image of a GFRα1^+^ A_al-16_ syncytium in a Het-cKO tubule at 8–9 weeks (P). Data are shown as mean ± SD. ^*^P < 0.05, ^**^P < 0.01. Statistical significance was assessed using an unpaired two-tailed Student t-test for (B–D, F–H, J, L); an unpaired two-tailed Welch t-test was used for (O); one-way ANOVA with Dunnett multiple comparisons test for (N). Scale bars, 100 μm (E); 20 μm (I, K, M, P).

To generate germline-specific *Lamc1* cKO mice, the *Lamc1^flox^* allele was recombined using the *Vasa-Cre* transgene. To minimize the risk of ectopic whole-body recombination, the *Vasa-Cre* transgene was maintained exclusively through paternal inheritance [36, 37]. We initially analyzed germline-specific heterozygous cKO mice (*Vasa-Cre^Tg^; Lamc1^flox/+^*; Het-cKO) and *Lamc1^flox/+^* mice (Control), as shown in Figure 2. Due to the high recombination efficiency of *Vasa-Cre^Tg^* in the male germline, homozygous *Lamc1* cKO mice (*Vasa-Cre^Tg^; Lamc1^flox/flox^*) could not be generated through our breeding strategy (male *Vasa-Cre^Tg^; Lamc1^flox/+^* crossed with female *Lamc1^flox/flox^*). Instead, this crossing yielded only *Vasa-Cre^Tg^; Lamc1^flox/−^* mice as germline homozygous *Lamc1* cKO mice.

Because *Lamc1^flox/−^* mice reportedly exhibit hindlimb paralysis, due to reduced *Lamc1* expression in somatic tissues compared with *Lamc1*^+/−^ mice [34, 38], which can secondarily impair reproductive function, *Vasa-Cre^Tg^;Lamc1^flox/−^* mice were not used as the primary experimental model. Instead, they were analyzed as a supplementary experimental system to confirm loss of LAMC1 protein in GFRα1^+^ spermatogonia following Cre-mediated deletion of *Lamc1^flox^* allele (Figure S5) and assess whether homozygous germline deletion of *Lamc1* on a *Lamc1^flox/−^* background (*Vasa-Cre^Tg^; Lamc1^flox/−^*) exacerbates spermatogonial phenotypes compared with *Lamc1^flox/−^* control mice (Figure S6).

### *Lamc1* conditional knockout and pulse-labeling using the CreER-loxP system

**Figure 3:**
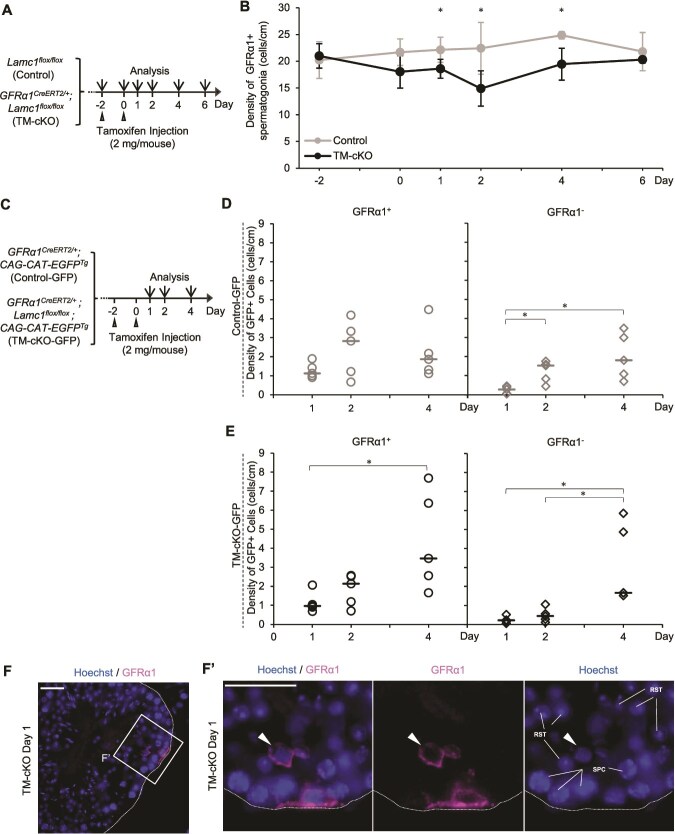
Phenotype of GFRα1^+^ spermatogonia in *GFRα1^CreERT2/+^; Lamc1^flox/flox^* mouse testes. (A) Experimental schedule for tamoxifen-induced *Lamc1* deletion in GFRα1^+^ spermatogonia. Tamoxifen (2 mg/mouse) was administered to male *Lamc1^flox/flox^* (Control) and *GFRα1^CreERT2/+^; Lamc1^flox/flox^* (TM-cKO) mice twice at 2-day intervals. Testes were collected on days −2, 0, 1, 2, 4, and 6, with the last day of tamoxifen injection designated as day 0. (B) Relative density of GFRα1^+^ spermatogonia along the perimeter of seminiferous tubules in the control and TM-cKO testes over time. A 90-tubule cross-section was analyzed per animal (*n* = 5 mice for each time point). An unpaired two-tailed Welch t-test was used to compare Control and TM-cKO values at each time point. (C) Experimental schedule for lineage tracing. Tamoxifen (2 mg/mouse) was administered to male *GFRα1^CreERT2/+^; CAG-CAT-EGFP^Tg^* (Control-GFP) and *GFRα1^CreERT2/+^; Lamc1^flox/flox^; CAG-CAT-EGFP^Tg^* mice (TM-cKO-GFP) twice, at 2-day intervals. Testes were collected on days 1, 2, and 4, with the last day of tamoxifen injection designated as day 0. (D, E) Relative density of GFP^+^/GFRα1^+^ (left panel) and GFP^+^/GFRα1^−^ (right panel) cells along the perimeter of tubules of Control-GFP (D) and TM-cKO-GFP (E) mice. Individual data points are plotted, with horizontal lines indicating the median. Two hundred seventy tubule cross-sections were analyzed per animal (*n* = 5 mice for each time point). Statistical significance was determined using the Kruskal–Wallis test, followed by the Steel–Dwass exact test for all pairwise comparisons among Day 1, Day 2, and Day 4. No significant difference in the density of GFP^+^/GFRα1^+^ cells was detected in Control-GFP testes by the Kruskal–Wallis test (*P* > 0.05). For GFP^+^/GFRα1^−^ cells in Control-GFP testes and for both GFP^+^/GFRα1^+^ and GFP^+^/GFRα1^−^ cells in TM-cKO-GFP testes, statistical significance was determined by the Kruskal–Wallis test (*P* < 0.05), and pairwise differences identified by the Steel–Dwass exact test are indicated by asterisks. (F, F’) Example of a GFRα1^+^ cell detached from the basement membrane in the TM-cKO testis at day 1 (arrowhead), while another GFRα1^+^ cell remains attached. White dotted lines indicate the basement membrane. Magenta, GFRα1; blue, hoechst33342. Scale bars, 20 μm. Data are shown as the mean ± SD. ^*^P < 0.05.

For tamoxifen-inducible *Lamc1* cKO with or without GFP pulse-labeling, and their corresponding controls, the following mouse lines were used: *GFRα1^CreERT2/+^; Lamc1^flox/flox^* (TM-cKO), *Lamc1^flox/flox^* (Control), *GFRα1^CreERT2/+^; Lamc1^flox/flox^; CAG-CAT-EGFP^Tg^* (TM-cKO-GFP)*,* and *GFRα1^CreERT2/+^; CAG-CAT-EGFP^Tg^* (Control-GFP), as shown in Figure 3. Tamoxifen (Nacalai Tesque, Kyoto, Japan) was dissolved in sesame oil (Nacalai Tesque) and administered intraperitoneally to 8–9-week-old mice at a dose of 2 mg per mouse twice with a 2-day interval. CreER-mediated recombination occurs during the first ~2 days after tamoxifen administration. Under this regimen, the exact recombination efficiency at the *Lamc1^flox^* locus could not be quantified in vivo due to the absence of a locus-specific reporter. The *CAG-CAT-EGFP* reporter genes coexisting with the *Lamc1^flox^* gene in the same nucleus showed approximately 10% recombination efficiency in control-GFP and TM-cKO-GFP testes (the proportion of GFP^+^ cells among total GFRα1^+^ cells two days after the final tamoxifen injection), confirming effective CreER activation in at least 10% of GFRα1^+^ spermatogonia in these mice.

### Single-cell RNA-seq analysis

Read count matrix and clustering list of single-cell RNA-seq data from adult mouse testis were obtained from Green et al. 2018 (GSE112393) [5]. Read counts were normalized to counts per 10,000 cells, and gene expression levels were defined by subsequent log-transformation (log_e_​[value+1]). The mean expression level for each cluster was then calculated using R (version 4.2.3) (R Core Team, 2023).

### Histological analyses

Mouse testes were fixed by immersion overnight in either Bouin solution (Wako Pure Chemical Industries, Osaka, Japan) or 4% paraformaldehyde (PFA; Nacalai Tesque) in phosphate-buffered saline (PBS; Nacalai Tesque). The tissues were embedded in paraffin and then sectioned at 4 μm thickness. The sections derived from Bouin fixation were subjected to Periodic-acid-Schiff (PAS) staining and counterstained with hematoxylin, while sections fixed in 4% PFA-PBS were stained with hematoxylin and eosin. Evaluation of the cycle of seminiferous tubules was performed as described previously [39]. Histological analyses were performed using upright microscopes (BX53; Evident, Tokyo, Japan) equipped with a CCD camera (DP73 and DP71; Evident).

### Immunofluorescent staining of tissue sections

For immunofluorescent staining, isolated mouse testes were fixed with 4% PFA-PBS. Subsequently, they were soaked in 10% and 20% (w/v) solutions of sucrose (Nacalai Tesque) in PBS for 30 min and overnight, respectively, at 4°C. Samples were then cryo-embedded in an optimal cutting temperature compound (Sakura Finetek Japan, Tokyo, Japan), sectioned at 10-μm using a CM1520 cryostat (Leica, Wetzlar, Germany), dried for 30 min, and washed twice in PBS at 4°C for 20 min. Sections were blocked for 1 h at room temperature using a solution of 4% donkey serum (Sigma–Aldrich, St. Louis, MO, USA: D9663) in Blocking One (Nacalai Tesque). Primary antibodies were incubated overnight at 4°C in 1:1 PBS/Blocking One with anti-GFRα1 (1∶1000 dilution; R&D Systems, Minneapolis, MN, USA: AF560), anti-Lam1 + 2 (1:500 dilution; Abcam: ab7463), anti-PLZF (1:1000 dilution; R&D Systems: AF2944), anti-Rarg (1:500 dilution; Cell Signaling Technology, Danvers, MA, USA: #8965), anti-PH3 (1:500 dilution; Merck, Darmstadt, Germany: 06-570), anti-cKit (1:1000 dilution; R&D Systems: AF1356), anti-Laminin gamma 1 clone A5 (1:50 dilution; Sigma Aldrich, Burlington, MA, USA: MAB1914P), and anti-GFP (1:1000 dilution; Abcam: ab13970). After three 10-min washing in PBST (0.1% Tween-20 [Nacalai Tesque] in PBS), the sections were incubated with secondary antibodies for 1 h at room temperature. The reaction was visualized with donkey anti-rabbit IgG Alexa Fluor 488 (1:400 dilution; Thermo Fisher Scientific, Waltham, USA: A-21206), donkey anti-goat IgG Alexa Fluor 594 (1:400 dilution; Thermo Fisher Scientific: A-11058), donkey anti-rabbit IgG Alexa Fluor 594 (1:400 dilution; Thermo Fisher Scientific: A-21207), donkey anti-goat IgG Alexa Fluor 488 (1:400 dilution; Thermo Fisher Scientific: A-11055), donkey anti-rat IgG Alexa Fluor 594 (1:400 dilution; Thermo Fisher Scientific: A-21209), donkey anti-chicken IgY Alexa Fluor 488 (1:400 dilution; Thermo Fisher Scientific: A78948), and Hoechst 33342 (1:5000; Nacalai Tesque: 19172-51). Observations and image acquisition were performed using a BX63 upright fluorescent microscope equipped with a DP74 CCD camera (Evident). Confocal images (Figure S5) were acquired using an FV3000 confocal laser scanning microscope equipped with a 100× oil immersion objective lens (Evident). For lineage tracing analysis, the presence or absence of GFRα1 expression in GFP-labeled cells was determined by double immunofluorescence for GFP and GFRα1. The density of each spermatogenic cell type was calculated as the number of cells per total perimeter of counted seminiferous tubule circumferences (cells/cm). Cell identification and counting were performed by visual inspection directly through the eyepieces using a fluorescent microscope equipped with a 60× water immersion objective lens (Evident), and the tubule perimeter was measured on digital images of the analyzed seminiferous tubules using ImageJ software (v1.53t, National Institutes of Health, Bethesda, MD, USA).

### Whole-mount immunofluorescent staining

Whole-mount seminiferous tubule immunofluorescent staining was performed as described previously [40], using anti-GFRα1 (1:1000 dilution; R&D systems: AF560), anti-FOXO1 (1:1000 dilution; Cell Signaling: #2880), anti-Cleaved Caspase3 (1:500 dilution; Cell Signaling: #9664), donkey anti-Goat IgG Alexa Fluor 488 (1:400 dilution; Thermo Fisher Scientific: A-11055), donkey anti-rabbit IgG Alexa Fluor 594 (1:400 dilution; Thermo Fisher Scientific: A-21207), donkey anti-goat IgG Alexa Fluor 594 (1:400 dilution; Thermo Fisher Scientific: A-11058), donkey anti-rabbit IgG Alexa Fluor 488 (1:400 dilution; Thermo Fisher Scientific: A-21206), and Hoechst 33342 (1:5000 dilution; Nacalai Tesque, 19172-51).

Observations were made and photographs were taken using a BX63 fluorescence microscope equipped with a DP74 CCD camera. Syncytial length of spermatogonia was determined by visually identifying continuous GFRα1 staining indicative of cell–cell connection, using a 60× water immersion objective lens. The total lengths of seminiferous tubules analyzed were as follows: For cleaved caspase-3 immunofluorescent staining and analysis of the relative frequency of GFRα1^+^ spermatogonial units (defined as spermatogonial categories classified by their connectivity state as A_s_, A_pr_ and A_al_), total tubule lengths of 5.96, 5.18, and 6.04 cm were analyzed in individual control mice (*n* = 3), and 4.81, 6.03, and 6.29 cm in individual Het-cKO mice (*n* = 3). For FOXO1 immunofluorescent staining, total tubule length of 4.71, 5.03, and 5.05 cm were analyzed in individual control mice (*n* = 3), 6.14, 6.18, and 5.11 cm in individual Het-cKO mice (*n* = 3), and 5.09, 5.21, and 5.79 cm in individual busulfan-treated mice (*n* = 3).

### Busulfan administration to induce germ cell depletion

Busulfan (Sigma–Aldrich) was prepared by dissolving in dimethyl sulfoxide (Nacalai Tesque) and diluting with an equal volume of sterile PBS [4]. To establish a germ cell depletion model for subsequent analysis of regeneration by surviving SSCs, mice were intraperitoneally injected once with Busulfan (10 mg/kg). Testes were collected at day 9, using the last day of Busulfan administration as reference day 0.

### Sperm counting

Sperm counting was performed as described previously [41, 42]. Briefly, spermatozoa were collected from the cauda epididymis, dispersed in human tubal fluid medium, and counted using a hemocytometer.

### Statistical analysis

Statistical analyses were performed using χ^2^ test, Fisher exact test, unpaired two-tailed Student t-test, unpaired two-tailed Welch t-test, Kruskal–Wallis test followed by the Steel–Dwass exact test, or one-way ANOVA followed by Dunnett multiple comparisons test (*P*-values are indicated in the figures). All statistical analyses were conducted using R version 4.2.3 (R Core Team, 2023), R version 4.5.2 (R Core Team, 2025) and KyPlot version 6.0.2 (KyensLab Inc.).

## Results

### Onset of laminin expression in GFRα1^+^ spermatogonia during sexual maturation

To understand the expression pattern of laminin in GFRα1^+^ spermatogonia and other types of surrounding cells, we performed immunofluorescent staining of testis sections using a pan-laminin antibody (Lam1 + 2), which broadly recognizes multiple laminin isoforms. In testis from 8-week-old adult mice, pan-laminin signals were consistently detected in the peritubular myoid cells and in PLZF^+^ A_undiff_, whereas it was absent in approximately 90% of c-Kit^+^ differentiating spermatogonia, as well as all spermatocytes and spermatids (Figure 1C–H).

We next examined the timing of laminin expression onset in GFRα1^+^ spermatogonia during postnatal stages. At 1 week of age, strong pan-laminin staining was evident in peritubular myoid cells (Figure 1I, J, and  S1A) and weak signal was detected in Sertoli cells (Figure 1I, K), while about 70% of GFRα1^+^ spermatogonia were negative for pan-laminin staining (Figure 1I, K, O). Between 1 and 4 weeks of age, the proportion of pan-laminin-positive GFRα1^+^ spermatogonia gradually increased (Figure 1O). By 8 weeks of age, all GFRα1^+^ spermatogonia exhibited cytoplasmic pan-laminin signal (Figure 1L, N, O), alongside the persistent and strong signal in myoid cells (Figure 1L, M and S1A), whereas the signal in Sertoli cells was undetectable (Figure S1B). The proportion of pan-laminin-positive c-Kit^+^ differentiating spermatogonia remained consistently low between 1 and 8 weeks of age (Figure S1C). These findings suggest that GFRα1^+^ spermatogonia express laminin in adulthood, with its expression initiated during sexual maturation.

### *Lamc1* expression in GFRα1^+^ spermatogonia in adulthood

To identify the laminin subunit genes expressed by GFRα1^+^ spermatogonia in the adult testis, we analyzed a previously published single-cell RNA sequencing dataset (GSE112393) [5]. GFRα1^+^ spermatogonia exhibited a laminin subunit gene expression profile, characterized by high levels of *Lama5*, *Lamb1*, *Lamb2*, and *Lamc1* (Figure 1P). The expression of these genes declined as the cells underwent differentiation during spermatogenesis (Figure S2A–D). Although the expression levels of each laminin subunit gene differed among GFRα1^+^ spermatogonia, Sertoli cells and myoid cells, *Lama5*, *Lamb1*, *Lamb2*, and *Lamc1* were commonly expressed between these cell types (Figure 1P). Given that laminin is generally secreted as a heterotrimer composed of one α, one β, and one γ subunit [28, 43–45], the expression profile of GFRα1^+^ spermatogonia, in which *Lama5* (*α5*), *Lamb1* and *Lamb2* (*β1* and *β2*), and *Lamc1* (*γ1*) are the most highly expressed members of their respective families, supports the notion that GFRα1^+^ spermatogonia mainly secrete laminin 511 and laminin 521, likely increasing the local laminin abundance in their vicinity.

### Germline-specific heterozygous deletion of *Lamc1* alters GFRα1^+^ spermatogonial behavior

Among the laminin subunits expressed in GFRα1^+^ spermatogonia, we focused on LAMC1, a core component shared across most laminin isoforms and essential for basement membrane formation during embryogenesis [28–30, 46]. To specifically assess germline-intrinsic functions of LAMC1 while preserving *Lamc1* expression in somatic cells including myoid cells and Sertoli cells, we initially analyzed germline-specific “heterozygous” cKO mice (*Vasa-Cre^Tg^; Lamc1^flox/+^*, hereafter Het-cKO) and control (*Lamc1^flox/+^*) mice (Figure 2A; see Method).

Recombination of the floxed allele in the germline was confirmed by genotyping offspring obtained from crosses between Het-cKO males and wild-type females. None of the 46 pups inherited the unrecombined *flox* allele (*flox/+*; 0/46), indicating a complete germline recombination. The offspring genotypes were distributed as *+/+* (*n* = 21) and *Δ/+* (*n* = 25), consistent with the expected 1:1 segregation ratio (χ^2^ test, χ^2^ = 0.3478, *P* = 0.5553). These results suggest that sperm carrying the *Δ* or *wild-type Lamc1* alleles possesses comparable fertilization and developmental potential (Figure S3A–C).

Body weight, testis weight, sperm count, seminiferous tubule morphology, and the distribution of seminiferous epithelial stages showed no significant differences between Het-cKO and control mice, except for body weight of 12 weeks of age (Figure 2B–E, Figure S4A and B). These results suggest that heterozygous *Lamc1* deletion does not affect gross spermatogenesis. The density of GFRα1^+^ spermatogonia remained comparable between Het-cKO and control testes throughout the period from 2 weeks to 7 months of age (Figure 2F). In addition, at 8 weeks of age, the densities of RARγ^+^ A_undiff_ and c-Kit^+^ differentiating spermatogonia showed no significant changes (Figure 2G and H). Despite the unaltered cell densities, GFRα1^+^ spermatogonia in Het-cKO testes exhibited a significantly higher proportion of phospho–Histone H3 (pH 3)^+^ cells, a marker of mitosis, compared to those in control testes (Figure 2I and J). In addition, the proportion of cleaved caspase-3^+^ cells, a marker of apoptosis, was also elevated in GFRα1^+^ spermatogonia in Het-cKO testes compared with that in control testes (Figure 2K and L). Furthermore, the fraction of GFRα1^+^ spermatogonia with nuclear localization of FOXO1, a reported marker of SSC quiescence [3], was significantly lower in Het-cKO testes than in controls, similar to the decline observed during busulfan-induced regeneration [3] (Figure 2M, and  N). Although not statistically significant, we observed a 5.4-fold increase in the proportion of GFRα1^+^ A_al-16_ among total GFRα1^+^ spermatogonial units in Het-cKO testes (0.276 ± 0.096%) compared with that in control testes (0.051 ± 0.003%) (Figure 2O and P). This tendency is consistent with a previous report describing transient increases of long GFRα1^+^ syncytia during SSC regeneration [4]. Together, these results suggest that heterozygous *Lamc1* deletion in germ cells reduces survival and increases proliferation of GFRα1^+^ spermatogonia, with these cells exhibiting characteristics of spermatogonia during regeneration.

To determine whether further reduction of germline *Lamc1* dosage exacerbates these phenotypes, we compared *Lamc1^flox/−^* and *Vasa-Cre^Tg^; Lamc1^flox/−^* mice (see Methods). Loss of LAMC1 protein in spermatogonia was confirmed in *Vasa-Cre^Tg^; Lamc1^flox/−^* mice (FigureS5). Although *Lamc1^flox/−^* mice exhibited hindlimb paralysis as previously reported [34, 38], testis weight and the densities of GFRα1^+^, RARγ^+^, and c-Kit^+^ spermatogonia did not differ significantly between the two genotypes, with the exception of an increase in GFRα1^+^ spermatogonial density at 8 weeks of age (Figure S6A–H). Notably, the proportions of pH3^+^, cleaved caspase-3^+^, and A_al-16_ GFRα1^+^ spermatogonia showed no significant differences between the two genotypes and were comparable to those observed in Het-cKO mice (Figure S6I–K). These results suggest that complete loss of LAMC1 in germ cells does not further exacerbate the alterations in GFRα1^+^ spermatogonial survival and proliferation beyond those observed following heterozygous deletion.

### Tamoxifen-induced *Lamc1* deficiency in GFRα1^+^ spermatogonia immediately leads to behavioral alteration

Given that the sparsely distributed GFRα1^+^ spermatogonia express laminin, we hypothesized that GFRα1^+^ spermatogonia-derived laminin autonomously and locally modulates spermatogonial behavior. If this is the case, a decrease in LAMC1 production in GFRα1^+^ spermatogonia would be expected to immediately induce detectable alterations in their behavior.

To test this hypothesis, we generated a tamoxifen-inducible GFRα1-CreER-based cKO mice carrying *Lamc1^flox/flox^* alleles (*GFRα1^CreERT2/+^; Lamc1^flox/flox^* mice; hereafter TM-cKO) and compared the density of GFRα1^+^ spermatogonia along the seminiferous tubules between TM-cKO and control (*Lamc1^flox/flox^*) mice over an 8-day period from the initial tamoxifen administration (Figure 3A). In control testes, the density of GFRα1^+^ cells remained essentially unchanged throughout the observation period following tamoxifen administration (Figure 3B). In contrast, in TM-cKO testes, GFRα1^+^ cell density was significantly reduced by day 2 and subsequently recovered by day 6 (Figure 3B). This suggests that reduced *Lamc1* expression in GFRα1^+^ cells immediately causes loss of GFRα1^+^ cells.

To investigate how *Lamc1* deletion in GFRα1^+^ spermatogonia affects the balance between proliferation and differentiation during the period of reduced GFRα1^+^ cell density in TM-cKO mice (Figure 3A and B), we performed short-term lineage tracing using GFP reporter mice (*GFRα1^CreERT2/+^; Lamc1^flox/flox^; CAG-CAT-EGFP^Tg^* [TM-cKO-GFP] and *GFRα1^CreERT2/+^; CAG-CAT-EGFP^Tg^* [Control-GFP]). In these mice, tamoxifen administration irreversibly labels GFRα1^+^ cells with GFP, which is retained through cell proliferation and after loss of GFRα1 expression. This strategy enabled us to characterize temporal changes in the densities of GFP-labeled GFRα1^+^ and GFRα1^−^ cell populations and thereby examine the balance between proliferation and differentiation of GFRα1^+^ cells within each genotype (Figure 3C). In control-GFP testes, the density of GFP-labeled GFRα1^+^ cells showed no significant difference over time (Figure 3D, left), whereas the density of GFP-labeled GFRα1^−^ cells increased significantly from day 1 to days 2 and 4 (Figure 3D, right), consistent with the maintenance of overall GFRα1^+^ cell density in control testes (Figure 3B). By contrast, in TM-cKO-GFP testes, the density of GFP-labeled GFRα1^+^ cells increased significantly from day 1 to day 4 (Figure 3E, left). The density of GFP-labeled GFRα1^−^ cells also increased significantly over time, with no significant difference between day 1 and day 2 but a significant increase between day 2 and day 4 (Figure 3E, right). These increases in both GFP-labeled GFRα1^+^ and GFRα1^−^ cell populations are consistent with the recovery phase of the overall GFRα1^+^ cell density in TM-cKO testes (Figure 3B). These results suggest that the initial reduction in GFRα1^+^ cell density in TM-cKO mice is followed by enhanced proliferation and a transiently altered balance between proliferation and differentiation of GFRα1^+^ cells.

Notably, although not statistically significant (*P* = 0.500 by Fisher exact test), we detected ectopic GFRα1^+^ cells localized in the lumen of the seminiferous tubules during days 1–2 in TM-cKO or TM-cKO-GFP testes (2/3600 tubules), whereas no such cells were observed in controls (0/3600 tubules) (Figure 3F and F’).

Together, these findings suggest that reduction of *Lamc1* expression in GFRα1^+^ spermatogonia rapidly alters their fate behavior within a few days, consistent with a model in which spermatogonia-derived laminin modulates their behavior by influencing the local microenvironment.

## Discussion

### Onset of laminin expression of GFRα1^+^ spermatogonia during sexual maturation

We found that GFRα1^+^ spermatogonia progressively acquire laminin expression during sexual maturation. Following birth, gonocytes migrate from the lumen to the basement membrane and give rise to GFRα1^+^ spermatogonia [47–50]. Despite such an attachment of GFRα1^+^ spermatogonia to the basement membrane, laminin expression in these cells remains minimal at this stage. Thus, the initial establishment of basement membrane contact by GFRα1^+^ spermatogonia does not coincide with the onset of laminin expression, suggesting that basement-membrane contact alone does not account for its induction and that additional maturation-associated signals are required.

The increase in the number of GFRα1^+^ spermatogonia that express laminin coincides with the progressive maturation of the testicular environment over the first 6 postnatal weeks. During this period, Sertoli cells cease proliferation by 2 weeks, mature and form the blood–testis barrier by 3 weeks [51–53]. Meanwhile, germ cells complete the first wave of spermatogenesis, and the seminiferous tubules expand to their mature size by approximately 6 weeks, supporting steady-state spermatogenesis [54–56]. Notably, between 2–4 postnatal weeks, the proportion of GFRα1^+^ cells expressing laminin rises to nearly 100%, coinciding with a sharp decline in their density, which then stabilizes (Figures 1O, 2F). These findings suggest that laminin expression marks a maturation-associated state transition in GFRα1^+^ spermatogonia, aligned with the establishment of steady-state spermatogenesis.

### Local extracellular matrix modulation by GFRα1^+^ spermatogonia via laminin expression

We found that GFRα1^+^ spermatogonia express *Lama5, Lamb1, Lamb2,* and *Lamc1* in adult testes. Immunoelectron microscopy in rats has previously demonstrated the presence of laminin within intracellular vesicles of spermatogonia [31], supporting the idea that GFRα1^+^ spermatogonia secrete laminin-511 and -521. Laminin-511 and -521 are among the least characterized laminin isoforms [25, 29], and their physiological roles in the testis are not well understood. Since GFRα1^+^ spermatogonia are vastly outnumbered in cell density by peritubular myoid cells, which are the major source of testicular laminin [31], the total amount of laminin derived from GFRα1^+^ cells likely contributes little to the overall structural integrity of the basement membrane. Indeed, consistent with previous reports showing that *Lamc1^+/−^* mice are fertile [46], our study showed that germline-specific heterozygous deletion of *Lamc1* did not affect the gross architecture of seminiferous tubules.

Focusing instead on the local microenvironment of GFRα1^+^ spermatogonia, the localized accumulation of laminin-511 and -521 can be more significant for GFRα1^+^ spermatogonia than for overall seminiferous tubule structure. Given that both peritubular myoid cells and GFRα1^+^ spermatogonia produce laminin-511 and -521, GFRα1^+^ cell-derived laminins likely do not act as a distinct molecular cue but rather enhance the pericellular laminin concentration around GFRα1^+^ spermatogonia. Although laminin produced by GFRα1^+^ spermatogonia is most likely incorporated into the basement membrane, it is also possible that GFRα1^+^ cell-derived laminin transiently forms a pericellular matrix that remains associated with their cell surface through polymerization and receptor binding [30], thereby regulating their spatial localization and cellular behavior. Due to current technical limitations, we could not directly evaluate the secretion, distribution, and turnover of GFRα1^+^ cell-derived laminin. Future advances in laminin live-imaging technologies will be required to elucidate the in vivo dynamics and functional roles of laminin derived from these cells.

### Self-producing laminin supports the behavior of GFRα1^+^ spermatogonia

The two cKO mouse models provide genetic evidence that laminin produced by GFRα1^+^ spermatogonia modulates their own survival and behavior. In the Het-cKO model, GFRα1^+^ spermatogonia exhibit reduced survival accompanied by compensatory proliferation, whereas the TM-cKO model demonstrates a rapid loss of GFRα1^+^ spermatogonia following the deletion of laminin, as discussed below.

In the first model, Het-cKO mice, heterozygous deletion of *Lamc1* in all germ cells led to increased cell death of GFRα1^+^ spermatogonia, accompanied by elevated proliferation, reduced nuclear FOXO1 levels, and an increased frequency of long syncytia (Figure 2I–P). Although *Lamc1^+/−^* mice have been reported to be fertile [46], our detailed analysis of GFRα1^+^ spermatogonia revealed a previously unrecognized defect in their behavior. These regenerative hallmarks likely represent compensatory proliferation in response to increased cell loss, thereby maintaining GFRα1^+^ spermatogonial density and steady-state spermatogenesis in Het-cKO mice. Notably, this phenotype closely resembled that observed following complete loss of LAMC1 in germ cells on a somatic-cell *Lamc1^flox/−^* background (*Vasa-Cre^Tg^*; *Lamc1^flox/−^* mice; Figure S6), consistent with the notion that *Lamc1* is haploinsufficient in germ cells for regulating spermatogonial survival and behavior. However, because *Lamc1* deletion occurred broadly in the germline in this model, the specific contribution of *Lamc1* expression within GFRα1^+^ spermatogonia themselves needed to be evaluated.

To address this issue, we employed a second model, TM-cKO, in which *Lamc1* deletion was induced specifically in adult GFRα1^+^ spermatogonia. In this model, tamoxifen-induced CreER-mediated recombination of one or both *Lamc1* alleles in a subset of GFRα1^+^ spermatogonia caused a rapid cell loss of GFRα1^+^ spermatogonia, followed by recovery of cell density (Figure 3A and B). This initial loss could, theoretically, result from either cell death or enhanced differentiation. Pulse-labeling experiments using TM-cKO-GFP mice demonstrated that GFRα1^+^ spermatogonia produced only a few GFRα1^−^ differentiating progeny by day 2 (Figure 3E), indicating that the initial loss primarily reflected cell death rather than differentiation. By day 4, the increase of GFP-labeled GFRα1^+^ and GFRα1^−^ cells suggested increased proliferation of GFRα1^+^ spermatogonia, accompanied by an imbalance between proliferation and differentiation (Figure 3E). This fate behavior accounts for the recovery of GFRα1^+^ cell density observed in TM-cKO testes during the same period (Figure 3B). While the increased proliferation of GFRα1^+^ spermatogonia is consistent with a secondary regenerative response to the initial cell loss, the very rapid emergence of this phenotype within a few days following cKO raises the possibility that laminin deficiency may also exert a direct, primary effect on the proliferative behavior of GFRα1^+^ spermatogonia.

During the period of initial cell loss, we also observed a rare event in which a small fraction of GFRα1^+^ spermatogonia detached from the basement membrane and migrated toward the lumen (Figure 3F and F'), a phenotype not previously described in the studies of mammalian SSCs. The frequency of this detachment phenotype was not statistically significant and did not account for the overall reduction in GFRα1^+^ cell density. Nevertheless, the occurrence of this phenotype implies that laminin contributes to the physiological fail-safe retention of GFRα1^+^ spermatogonia within the basal compartment of seminiferous tubules. The rapid emergence of multiple phenotypes (reduced survival, increased proliferation, imbalance of proliferation and differentiation, detachment from the basement membrane, and ectopic migration toward the lumen) in TM-cKO mice, together with the limited diffusibility of laminin [57], which confines its distribution to the immediate vicinity of the secreting cell, suggests that laminin derived from GFRα1^+^ spermatogonia modulates spermatogonial survival and behavior in a cell-autonomous, locally acting manner.

Future studies combining short-term behavioral analysis of GFRα1^+^ spermatogonia after laminin cKO with gene expression profiling will be important for elucidating how self-produced laminin is sensed by these cells and how downstream signaling pathways regulate their survival and behavior.

### A conserved mechanism for dynamic microenvironment formation in migrating tissue stem cells

Aside from the germline, a similar cell-intrinsic mechanism has been observed in the skeletal muscle stem cell system in mice, where activated stem cells transiently secrete fibronectin onto the basement membrane during regeneration to regulate stem cell pool expansion [58]. In skeletal muscle, where the timing and location of regeneration are unpredictable, such stem cell-mediated ECM remodeling provides flexible microenvironmental control, likely contributing to the robustness of stem cell regenerative capacity. In contrast, mouse GFRα1^+^ spermatogonia, including SSCs, require constant regulation during their continuous migration under homeostatic conditions. By analogy, GFRα1^+^ spermatogonia may employ a similar self-tuning mechanism to reconcile their continual migration with precise behavioral regulation within an open niche. Thus, a dynamic and self-tuned microenvironment may represent a conserved principle of tissue stem cell regulation. Whether this principle can extend to the germline of other mammalian species remains unknown, and resolving this question will be essential for understanding its significance in germ cell biology.

## Conclusion

In conclusion, mouse GFRα1^+^ spermatogonia modulate their survival and behavior through laminin expression, likely by influencing the local ECM microenvironment around GFRα1^+^ spermatogonia. Such cell-autonomous regulation allows GFRα1^+^ spermatogonia, including SSCs, to form an appropriate local microenvironment within the testicular open niche, supporting stable behavior of spermatogonia during spermatogenesis.

## Supplementary Material

Kawabe_et_al_Suppl_s_rev_submit_ioag032

## Data Availability

Any additional data are available from the corresponding author upon reasonable request.
