# Supplementary material for: Undifferentiated spermatogonia modulate their behavior via the expression of basement membrane protein laminin
Source: Biol Reprod. 2026 Feb 3;114(4):1507–19. doi: 10.1093/biolre/ioag032 (PMC13079447; doi:10.1093/biolre/ioag032)
Supplement: Kawabe_et_al_Suppl_s_rev_submit_ioag032 [file kawabe_et_al_suppl_s_rev_submit_ioag032.pdf]

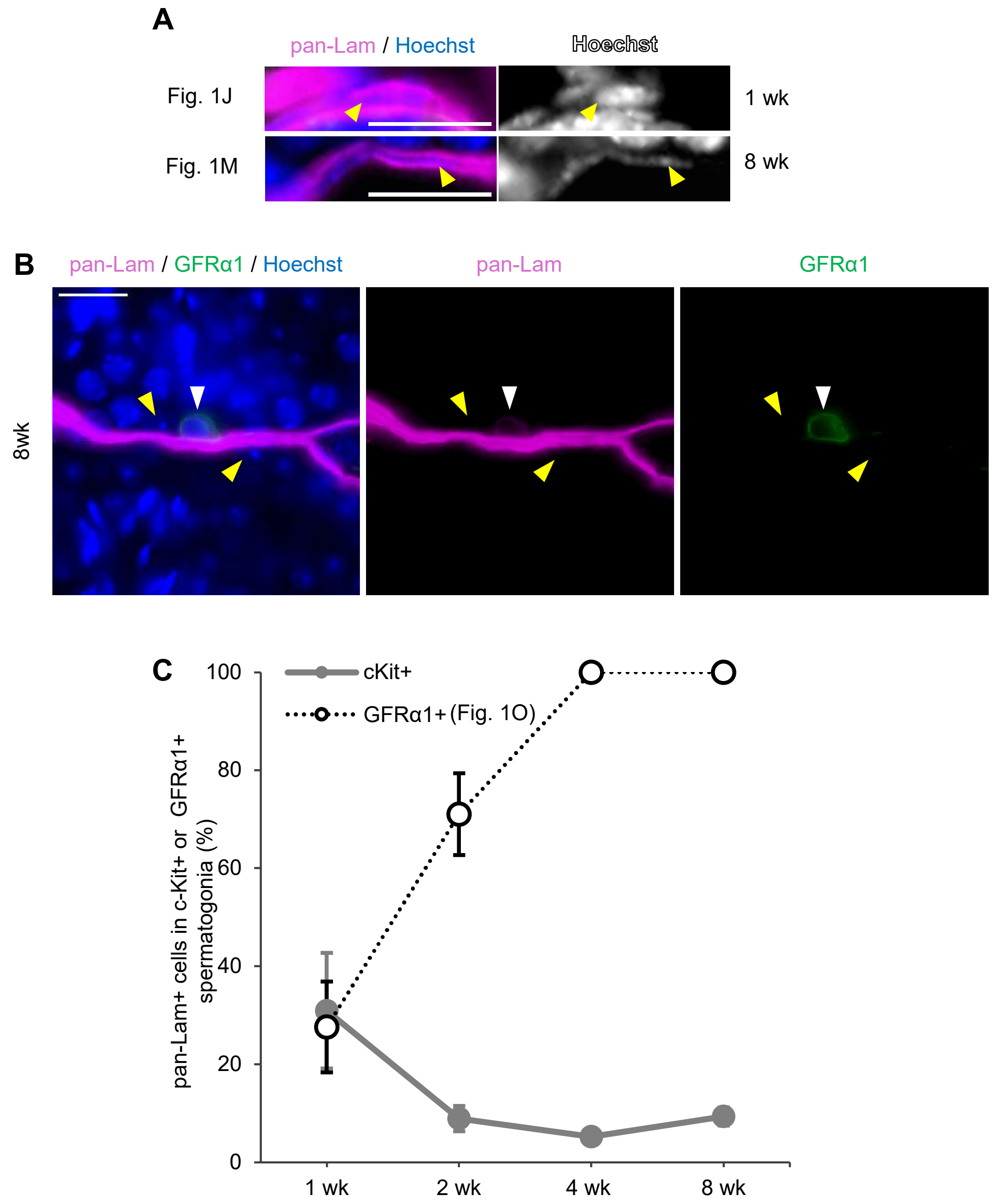

Fig. S1 Laminin expression in peritubular myoid cells and c-Kit⁺ spermatogonia.
(A) Representative immunofluorescence images of peritubular myoid cells. Left panels show merged images of pan-Laminin (magenta) and Hoechst (blue), and right panels show grayscale Hoechst staining. Merged images are reproduced from Figure 1J and M to demonstrate the presence and morphology of myoid cell nuclei. Yellow arrowheads indicate peritubular myoid cell nuclei, which appear flattened and are located adjacent to the cytoplasmic Laminin signals. **(B)** Representative immunofluorescence images of Sertoli cells. Left panel shows merged image of pan-Laminin (magenta), GFRα1 (green) and Hoechst (blue). Middle panel shows image of pan-Laminin staining. Right panel shows image of GFRα1 staining. Yellow arrowheads indicate Sertoli cells, which lack detectable pan-Laminin signals, whereas the white arrowhead indicates a GFRα1^{+} spermatogonium exhibiting cytoplasmic pan-Laminin staining. **(C)** Percentage of pan-Laminin^{+} cells among c-Kit^{+} spermatogonia at 1, 2, 4, and 8 weeks of age. The dotted line represents data for GFRα1^{+} spermatogonia, re-plotted from Figure 1O for comparison. c-Kit^{+} spermatogonia were counted in 50 seminiferous tubule sections per mouse (n = 3 mice at each time point). Data are presented as mean ± SD. Scale bars, 20 μm.

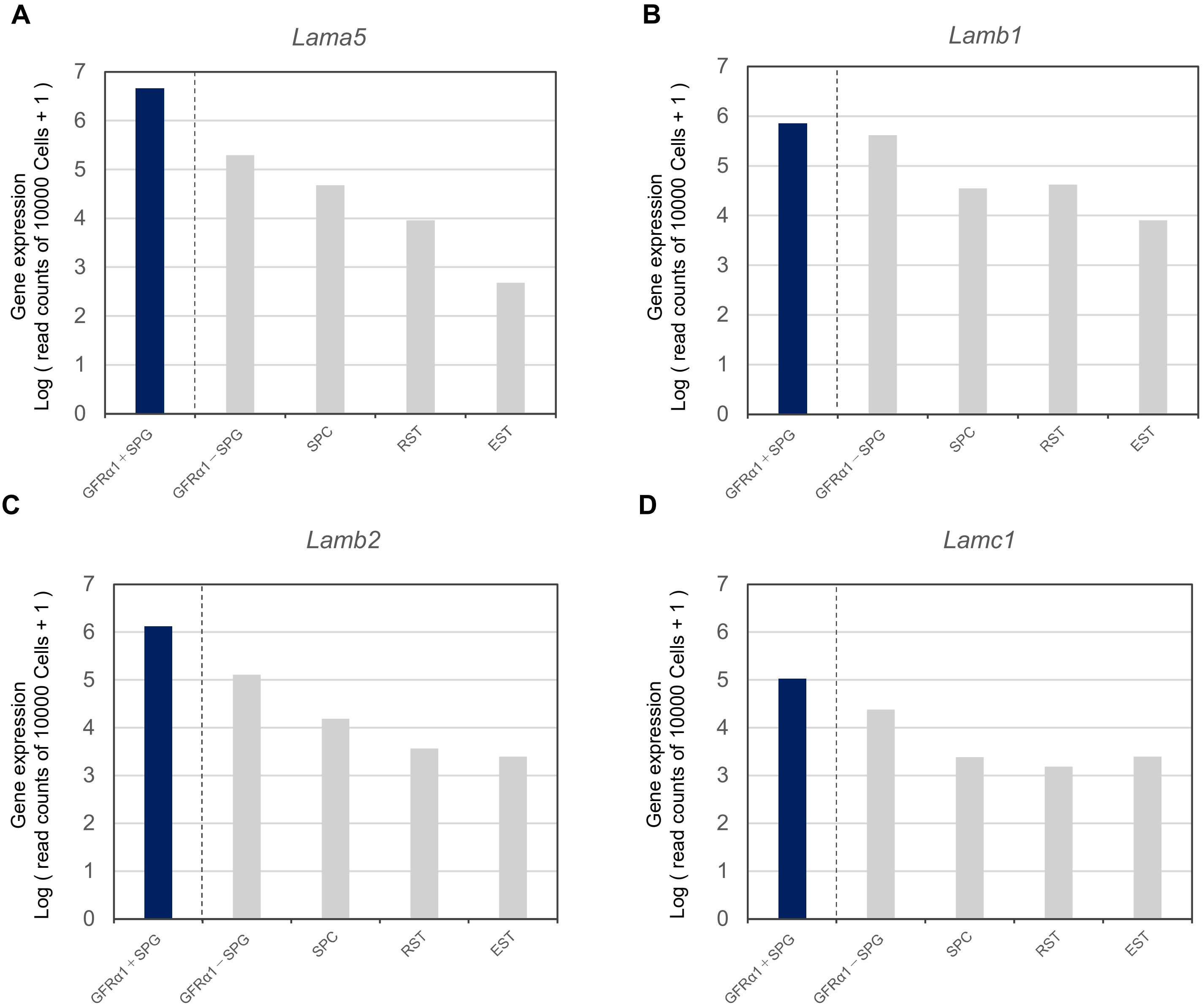

Fig. S2 Gene expression of Laminin subunits in male germ cells.

(A-D) Bar graphs showing high expression of Laminin subunits (*Lama5, Lamb1, Lamb2, Lamc1*) in GFRα1-positive spermatogonia (GFRα1+ SPG) compared to other germ cell types in adult mouse testes. Data was obtained from a single-cell RNA-seq dataset (Green et al., 2018, ref [38]). Other cell types include GFRα1-negative spermatogonia (GFRα1− SPG), spermatocytes (SPC), round spermatids (RST), and elongated spermatids (EST).

A

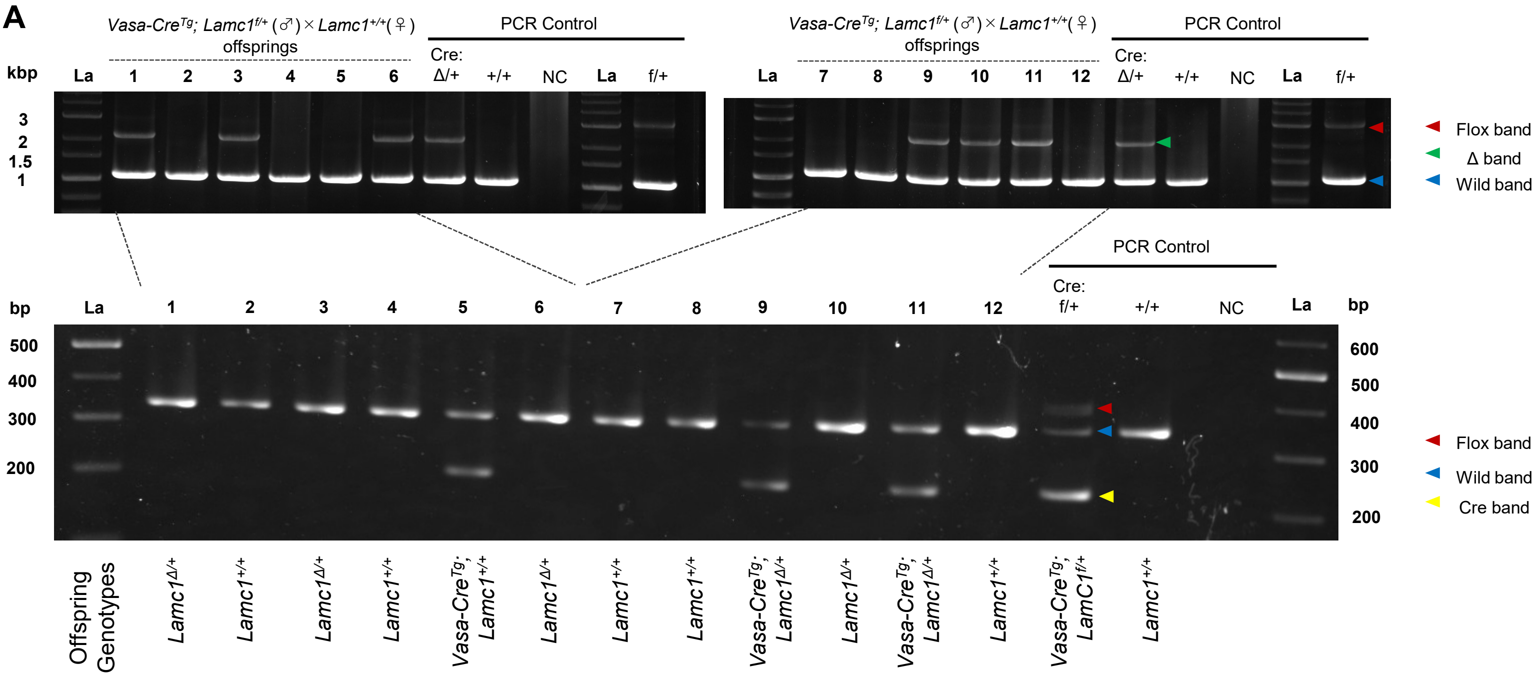

B

Mating combination	Offspring Genotype	Pups Number	Percentage (%)	Expected Percentage (%)	Cre status	Pups Number	Each Genotype Cre Percentage (%)	Expected Percentage (%)
Lamc1$^{flox/+}$ (♂, N=3)	**f/+**	**19**	**48.7**	**50.0**	Cre −	19	100.0	100.0
					Cre +	0	0.0	0.0
×	**Δ/+**	**0**	**0.0**	**0.0**	Cre −	0	0.0	0.0
					Cre +	0	0.0	0.0
Lamc1$^{+/+}$ (WT: ♀, N=6)	**+/+**	**20**	**51.3**	**50.0**	Cre −	20	100.0	100.0
					Cre +	0	0.0	0.0
	**Total**	**39**	**100.0**	**100.0**				

C

Mating combination	Offspring Genotype	Pups Number	Percentage (%)	Expected Percentage (%)	Cre status	Pups Number	Each Genotype Cre Percentage (%)	Expected Percentage (%)
Vasa-Cre^{Tg}; *Lamc1*$^{flox/+}$ (♂, N=3)	**f/+**	**0**	**0**	**0.0**	Cre −	0	0.0	0.0
					Cre +	0	0.0	0.0
×	**Δ/+**	**25**	**54.3**	**50.0**	Cre −	13	52.0	50.0
					Cre +	12	48.0	50.0
Lamc1$^{+/+}$ (WT: ♀, N=8)	**+/+**	**21**	**45.7**	**50.0**	Cre −	11	52.4	50.0
					Cre +	10	47.6	50.0
	**Total**	**46**	**100.0**	**100.0**				

Fig. S3 Breeding scheme to assess germline recombination efficiency

(A) PCR genotyping results showing the detection of wild-type (+), floxed (flox), and deleted (Δ) alleles. PCR products are wild-type (1.1 kbp), flox (3.0 kbp), and Δ (2.1 kbp). The presence of the Vasa-Cre transgene was determined using Cre-specific primers (Cre-Tg: 240 bp; wild-type: 337 bp; flox: 371 bp).
(B, C) Tables summarizing the number and percentage of offspring with each genotype and Cre status. The '+/Δ' genotype indicates successful germline excision. Expected Mendelian ratios are included for comparison.

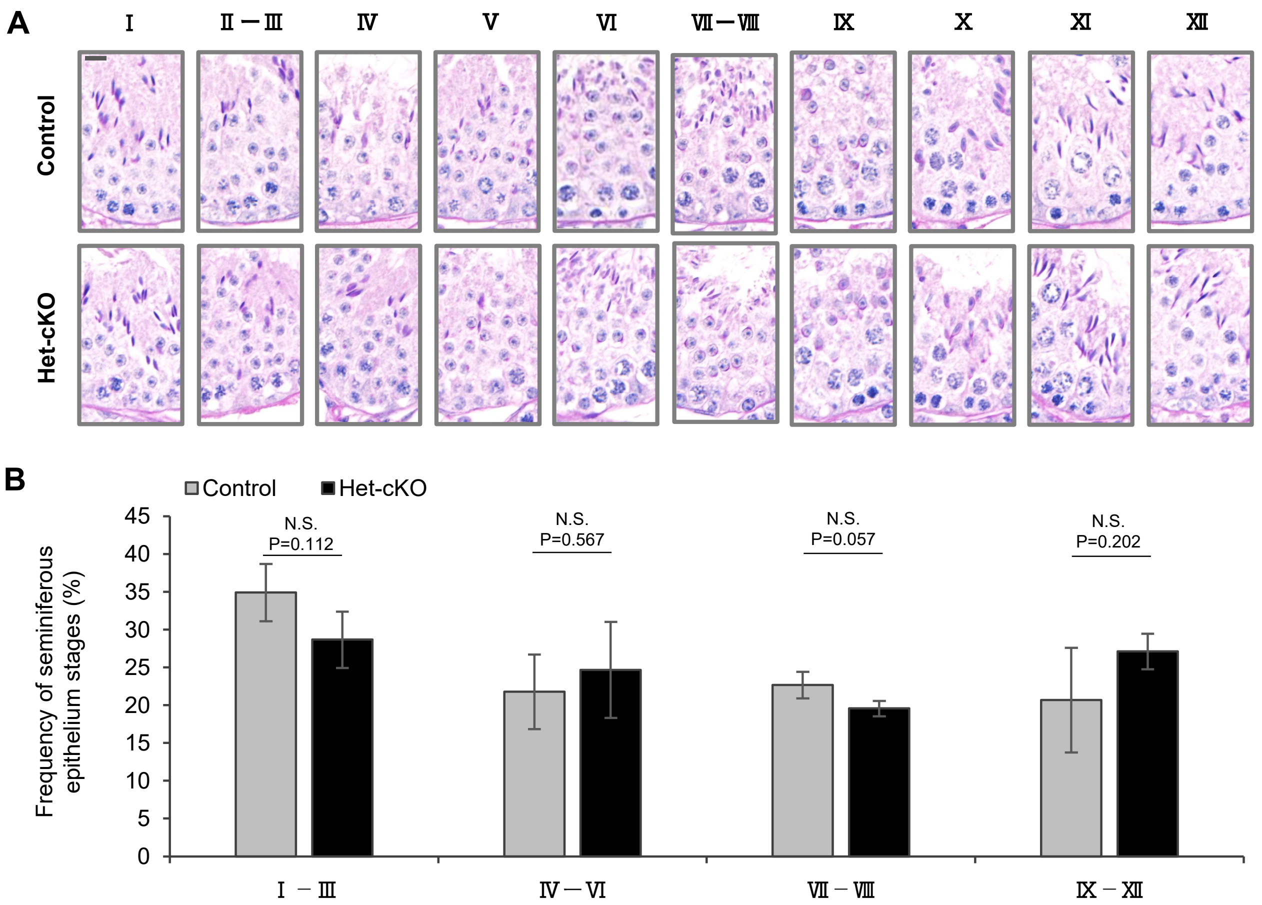

Fig. S4 The cycle of seminiferous epithelium in *Vasa-Cre*^{Tg}*; Lamc1*$^{flox/+}$ mouse testes.

(A) PAS-hematoxylin-stained cross-sections of Control and Het-cKO mouse testes at 8-9 weeks of age. Each stage (I-XII) of the seminiferous epithelial cycle is indicated. Scale bar = 10 μm.

(B) Quantification of seminiferous tubules per stage from the sections in (A). The number of seminiferous tubules was scored in 150 tubules per animal (n = 3). Data are shown as the mean ± SD. Statistical significance was determined by unpaired two-tailed Student's t-test. *P < 0.05; N.S., not significant (P > 0.05).

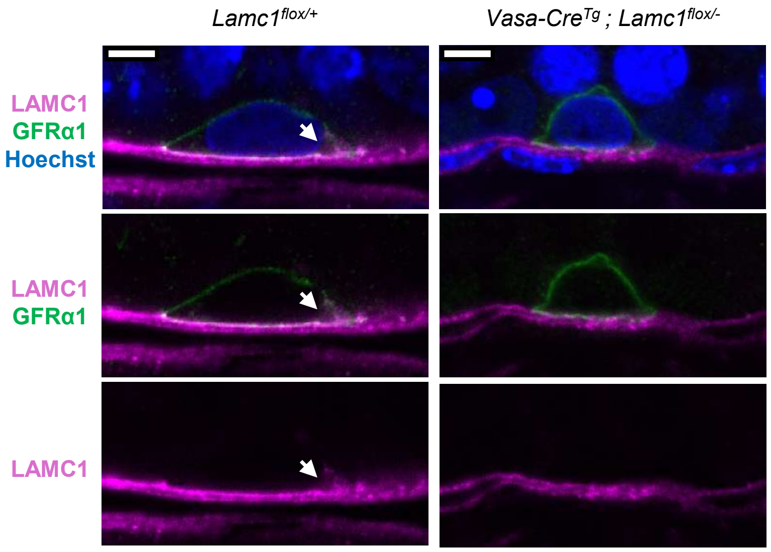

Fig. S5 Loss of LAMC1 expression in GFRα1⁺ spermatogonia of *Lamc1* germline homozygous knockout (*Vasa-Cre^{Tg} ; Lamc1$^{flox/-}$*) mice.

Representative immunofluorescence images of GFRα1^{+} spermatogonia in *Lamc1$^{flox/+}$* and *Vasa-Cre^{Tg}; Lamc1$^{flox/-}$* mouse testes at 8-9 weeks. Sections were stained for GFRα1 (green), LAMC1 (magenta), and Hoechst (blue). In the *Lamc1$^{flox/+}$* testis, the white arrows indicate LAMC1-positive signal in the cytoplasm of GFRα1^{+} spermatogonium. In the *Vasa-Cre^{Tg}; Lamc1$^{flox/-}$* mouse testis, LAMC1 signals was absent in the cytoplasm of GFRα1^{+} spermatogonium, whereas LAMC1 expression was retained in peritubular myoid cells. These results confirm cell-type-specific deletion of *Lamc1* in GFRα1^{+} spermatogonia. Scale bars, 5 μm

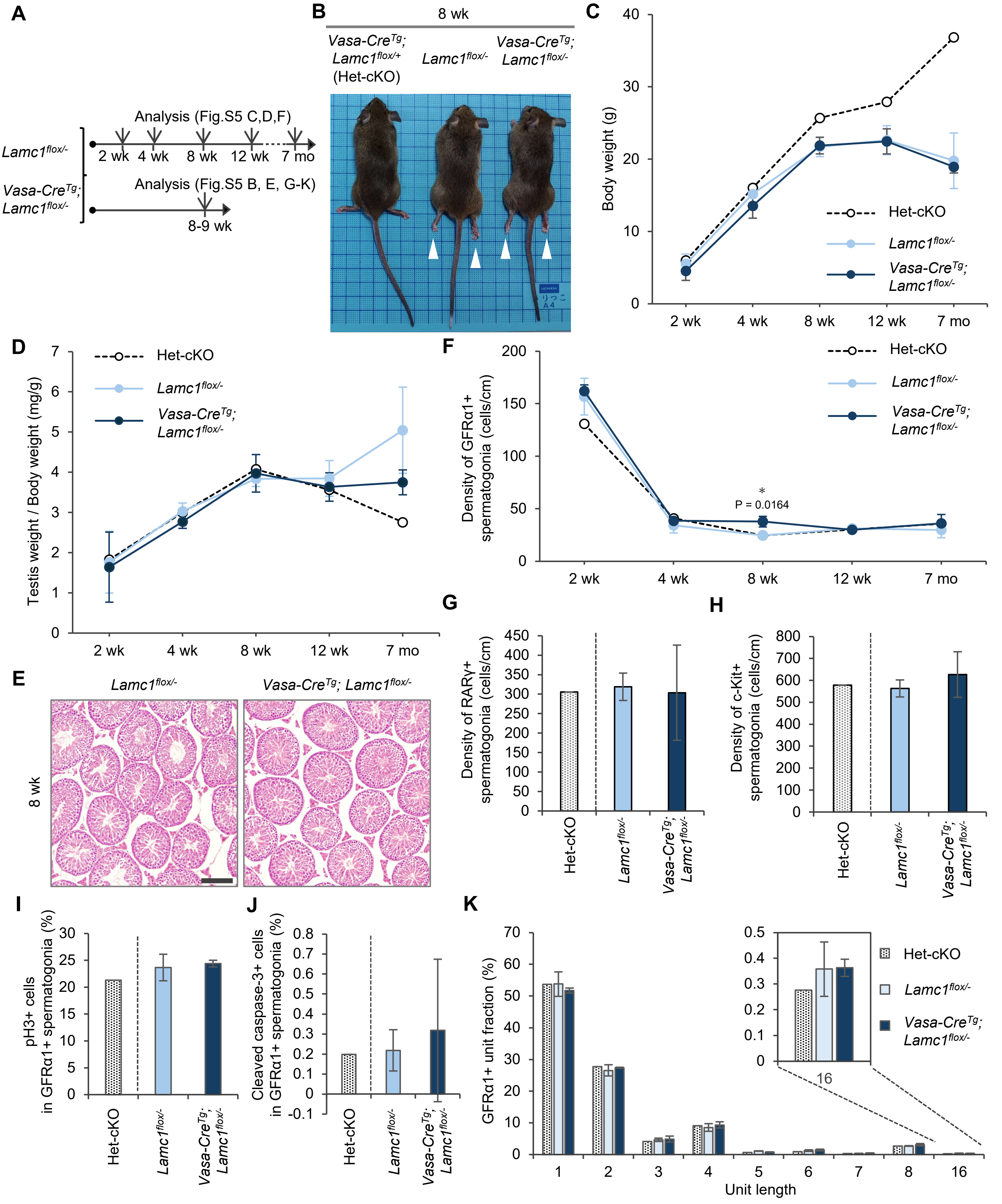

Fig. S6 Phenotypic analysis of *Vasa-Cre^{Tg}; Lamc1$^{flox/-}$ and Lamc1$^{flox/-}$* mouse testes

(A) Experimental schedule using *Vasa-Cre^{Tg}; Lamc1$^{flox/-}$* mice. **(B)** Gross appearance of mice at 8 weeks of age. Representative whole-body images of mice from each genotype are shown. White arrowheads indicate hind limb paralysis observed in *Lamc1$^{flox/-}$* and *Vasa-Cre^{Tg}; Lamc1$^{flox/-}$* mice. **(C, D)** Body weight (C) and testis weight normalized to body weight (D) in mice of each genotype at different ages. **(E)** Hematoxylin and eosin (H&E)-stained cross-sections of *Lamc1$^{flox/-}$* and *Vasa-Cre^{Tg}; Lamc1$^{flox/-}$* mouse testes at 8 weeks. **(F)** Relative density of GFRα1^{+} spermatogonia along the seminiferous tubule perimeter in mice of each genotype at different ages. GFRα1^{+} spermatogonia were counted in 50 seminiferous tubule sections per individual at 2 and 4 weeks, and in 90 sections at 8 and 12 weeks and 7 months (n = 3 individuals per time point). **(G, H)** Density of RARγ^{+} (G) and c-Kit^{+} (H) spermatogonia along the seminiferous tubule perimeter in each genotype at 8-9 weeks. RARγ^{+} and c-Kit^{+} spermatogonia were counted in 30 seminiferous tubule sections (n = 3 individuals). **(I)** Proportion of pH3^{+} cells among GFRα1^{+} spermatogonia in mice testes of each genotype. GFRα1^{+} spermatogonia were counted in 90 seminiferous tubule sections per individual (n = 3 individuals). **(J)** The percentage of cleaved caspase-3^{+} cells among GFRα1^{+} spermatogonia in mice of each genotype. **(K)** Frequency of GFRα1^{+} spermatogonia classified by syncytial length in each genotype testes at 8-9 weeks.

Data for *Lamc1$^{flox/-}$* and *Vasa-Cre^{Tg}; Lamc1$^{flox/-}$* are shown as mean ± SD. Data for *Vasa-Cre^{Tg}; Lamc1$^{flox/+}$* (Het-cKO) mice are re-plotted from Figure 2 for comparison (Data are shown as mean). Scale bar, 100 μm (E). Statistical analyses were performed between *Lamc1$^{flox/-}$* and *Vasa-Cre^{Tg}; Lamc1$^{flox/-}$* mice in (C, D, F-J), and for the frequency of A_{al-16} (Unit length 16) in (K), using an unpaired Student's t-test. *P < 0.05.
